# Sequential Injection Amperometric System Coupling with Bioreactor for In-Line Glucose Monitoring in Cell Culture Application

**DOI:** 10.3390/molecules27196665

**Published:** 2022-10-07

**Authors:** Chanyanut Wongsa, Suruk Udomsom, Apiwat Budwong, Kanokwan Kiwfo, Kate Grudpan, Pathinan Paengnakorn

**Affiliations:** 1 Biomedical Engineering Institute, Chiang Mai University, Chiang Mai 50200, Thailand; 2 Center of Excellence for Innovation in Analytical Science and Technology for Biodiversity-Based Economic and Society (I-ANALY-S-T_B.BES-CMU), Chiang Mai University, Chiang Mai 50200, Thailand; 3 Department of Chemistry, Faculty of Science, Chiang Mai University, Chiang Mai 50200, Thailand

**Keywords:** flow analysis, sequential injection, in-line sampling, in-line dilution, bioreactor, amperometry, screen-printed electrode, glucose, glucose oxidase

## Abstract

We proposed a specially designed sequential injection (SI) amperometric system coupling with a bioreactor for in-line glucose monitoring in cell culture. The system is composed of three main parts which are the bioreactor, SI system, and electrochemical detection unit. The bioreactor accommodates six individual cell culture units which can be operated separately under different conditions. The SI system enables automatic in-line sampling and in-line sample dilution, with a specially designed mixing unit; therefore, it has the benefits of fast analysis time and less contamination risk. The use of 3D-printed microfluidic components, a mixing channel, and a flow cell helped to reduce operational time and sample volume. A disposable screen-printed electrode (SPE), modified with glucose oxidase (GOD), carbon nanotube, and gold nanoparticle, was used for detection. The developed system provided a linear range up to 3.8 mM glucose in cell culture media. In order to work with cell culture in higher glucose media, the in-line sample dilution can be applied. The developed SI system was demonstrated with mouse fibroblast (L929) cell culture. The results show that glucose concentration obtained from the SI system is comparable with that obtained from the conventional colorimetric method. This work can be further developed and applied for in vitro cell-based experiments in biomedical research.

## 1. Introduction

Sequential injection (SI) analysis is a flow-based analysis technique that allows multiple samples to be manipulated separately in a segment controlled by a multi-position selective valve [1]. In past decades, a lot of SI systems have been developed and widely applied in various areas. Bioprocess monitoring is one of the SI applications that benefits from the in-line monitoring system [2]. The SI system allows a complex, multi-reagent, and multi-step chemical analysis, which is suitable for bioprocess including biopharmaceutical studies, fermentation bioreactor, and cell culture. Applications of the SI system to the biopharmaceutical process involving in vitro interaction with various types of cells have been previously reported; for example, monitoring of drug liberation [3,4], release test [5,6], and product quality [7].

SI has also been employed in fermentation bioreactors for in-line monitoring metabolites and products such as lactic acid, ammonia, glycerol, glucose, and formaldehyde [8,9]. In recent years, it has been applied to the mammalian cell culture in order to monitor conditions in a bioreactor and control product qualities. For cell culture application, the SI system has been combined with various analytical methods such as capillary electrophoresis [10], chromatography [11,12], and electrochemical analysis [13]. The SI systems have been beneficial for in-line determination in bioprocesses by allowing a short time process, automation, and multiple assays in one system [14].

Glucose is one of the major nutrients in cell culture media. Determination of glucose uptake rate in cell culture under experimental condition is necessary for cellular metabolism study. Therefore, an in-line monitoring system in a bioreactor can be beneficial for cell culture application. In a cell production, a change in glucose level is crucial for cell growth. The monitoring system can also be further developed into feedback control system to refresh culture media automatically.

Glucose sensors can be divided into several types depending on its transducer part of the sensor: electrochemical, optical, thermometric, piezoelectric, and magnetic. The major glucose sensor type is the electrochemical type because of its better sensitivity and reproducibility, easy maintenance, and low cost [15]. There have been numerous attempts to enhance the efficiency of glucose sensors by using nanomaterials, such as carbon nanotubes (CNTs), gold nanoparticles (AuNPs), platinum nanoparticles (PtNPs), etc., combined with glucose oxidase enzyme (GOD) coated on the electrode [16,17,18,19,20]. Some sensors have been applied to cell culture as shown in Table 1. Moreover, a couple of studies have reported the modification of glucose sensors advanced from a commercially available screen-printed electrode (SPE) [21,22].

In this work, a SI system coupled with bioreactor and amperometric detection was designed for in-line glucose monitoring in cell culture media. The bioreactor was modified from a previous report [28]; it was able to handle six individual cell culture units with different cell types or conditions up to six types/conditions at the same time. The SI system allowed programable sampling and adjustable in-line sample dilution for glucose analysis.

The amperometric detection unit utilized a commercial SPE modified with single-walled carbon nanotubes (SWCNTs), AuNPs, chitosan, and GOD. The application of the SI-bioreactor system with GOD–SWCNTs–AuNPs-modified SPE was demonstrated for the determination of glucose concentration in cell-cultured media from L929 cell line cultivation.

## 2. Results and Discussion

### 2.1. Performance of the Modified SPE

**Figure 1:**
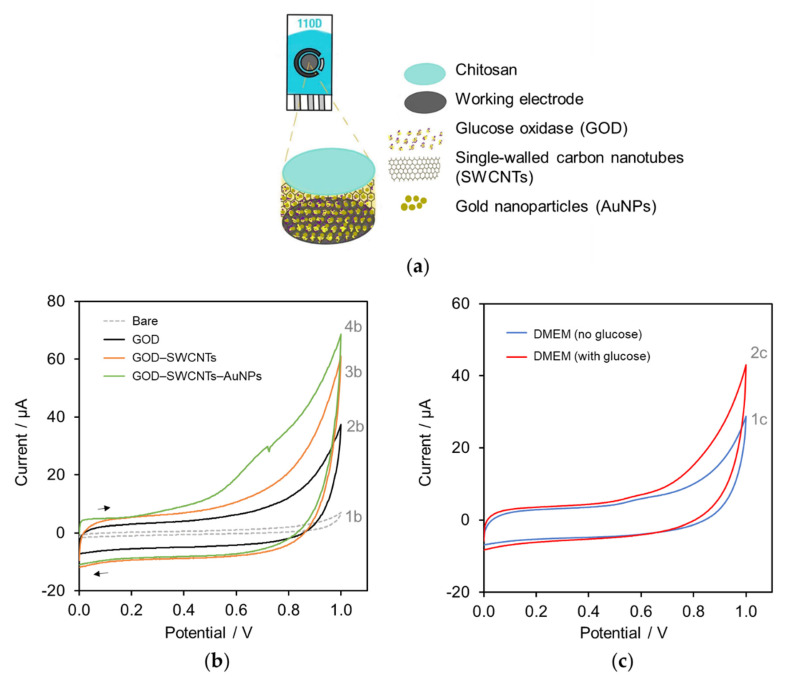
Modification of a SPE: (**a**) a schematic diagram showing GOD-AuNPs-SWCNTs-chitosan layer on the carbon working electrode surface (not to scale); (**b**) cyclic voltammograms of different modified electrodes; a bare commercial SPE (CV 1b), GOD-coated SPE (CV 2b), the mixing of GOD–SWCNTs-coated SPE (CV 3b), and the mixing of GOD–SWCNTs–AuNPs-coated SPE (CV 4b) in PBS; (**c**) cyclic voltammograms of GOD–SWCNTs–AuNPs-modified electrodes in DMEM in the presence of 10 mM glucose (CV 2c) and in the absence of glucose (CV 1c). Scan rate at 50 mV s^−1^.

For glucose determination in cell culture media, a carbon SPE was modified with a mixture of GOD, SWCNTs, and AuNPs. The GOD was immobilized on the working electrode surface together with the negatively charged SWCNTs and AuNPs to increase surface area and conductivity, while a chitosan provided a cationic 3D network covered on the electrode surface as illustrated in Figure 1a. Chitosan is a polycation natural polymer derived from chitin and a entangle network of chitosan can form hydrogel to immobilize enzyme and nanoparticles on the electrode surface. Amperometric determination of glucose was based on the activity of GOD as shown in Equations (1)–(3):

Glucose + GOD − FAD^+^ → Glucolactone + GOD − FADH_2_ (1)

GOD − FADH_2_ + O_2_ → GOD − FAD + H_2_O_2_ (2)

H_2_O_2_ → 2H^+^ + O_2_ + 2e^−^ (3)

The modified carbon SPE was characterized by cyclic voltammetry (CV). Figure 1b shows the comparison between a bare commercial SPE (CV 1b) and three different modified glucose sensors which are a GOD-coated SPE (CV 2b), the mixing of GOD–SWCNTs-coated SPE (CV 3b), and the mixing of GOD–SWCNTs–AuNPs-coated SPE (CV 4b). The result from CV of four different sensors in PBS shows an increase in charging current due to the electric double layer formed on the electrode interface increased when nanoparticles were incorporated onto the electrode surface, suggesting a change in electrical properties on the surface. It was found that GOD–SWCNTs–AuNPs-modified SPE exhibited higher current response compared with other three glucose sensors.

The GOD–SWCNTs–AuNPs-modified SPE was further examined in, instead of PBS, Dulbecco’s Modified Eagle Medium (DMEM) which is used in mammalian cell cultures for this work containing various nutrients, amino acids, vitamins, inorganic salts (e.g., CaCl_2_, KCl, and MgSO_4_), and carbonate buffer. It was found that the current obtained from DMEM (see Figure 1c, CV 1c) was lower than that obtained from the PBS medium (see Figure 1b, CV 4b). The observed phenomena were similar to the previous report [27]. It can be seen in Figure 1c that, for DMEM, the oxidative current due to glucose (CV 2c) was higher than that without glucose (CV 1c).

**Figure 2:**
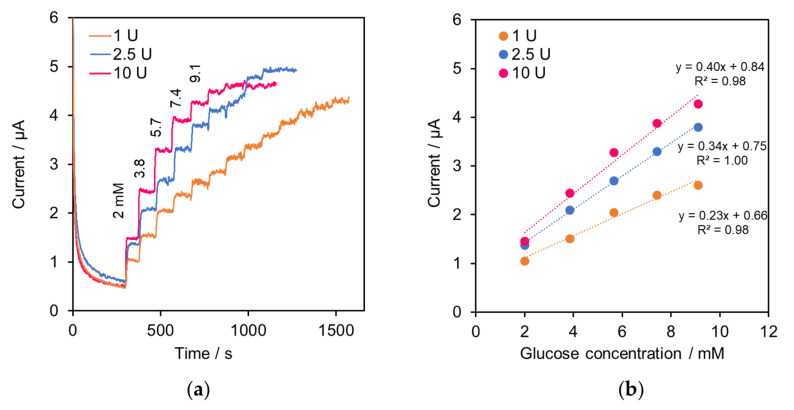
Optimization of GOD–SWCNTs–AuNPs-modified SPE: (**a**) a series of chronoamperograms of GOD–SWCNTs–AuNPs-modified SPE with various amount of GOD (1, 2.5 and 10 U) at applied potential +0.8 V showing increasing current response as glucose was added in PBS bulk solution; (**b**) plots between current response and added glucose concentration for each SPEs.

The GOD–SWCNTs–AuNPs modification of SPE was optimized by performing chronoamperometry with glucose in a bulk solution of PBS. The SPE were fabricated with various glucose oxidase enzyme amounts; 1, 2.5, and 10 U. Then, the amperometric measurement was conducted at applied potential +0.8 V. Figure 2a shows the amperometric response of three different sensors when the concentration of glucose increased in bulk solution. The relationship between the oxidative current and glucose concentration was plotted as the calibration curve shown in Figure 2b. The result shows that the current response increased linearly with glucose concentration in the working range up to 9.1 mM in all sensors. From this result, the sensor with 2.5 U GOD has compromised characteristics of sensitivity and proper working range to detect glucose compared with the others; hence, it was chosen to be employed as the detection unit for the SI system.

### 2.2. Characterization of the System for Determination of Glucose in Cell Culture Media

The performance characteristics of the system (see Section 3.1) for determination of glucose in culture media sample were investigated. The optimized sensor was assembled with the flow cell connected with the SI system. A series of glucose standard solutions with various concentrations were added to cell culture media (glucose-free DMEM), and each solution was sequentially transferred to the detection flow cell by an in-house syringe pump with a controlling program for the system calibration. The amperometric measurement was performed at applied potential +0.8 V.

**Figure 3:**
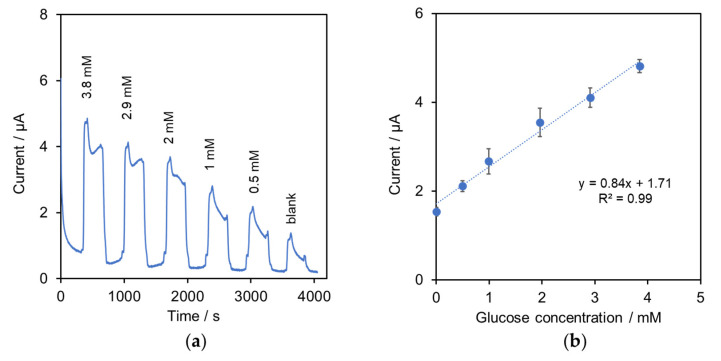
The system with the GOD–SWCNTs–AuNPs-modified SPE: (**a**) amperometric responses obtained from the system with various concentrations of glucose in DMEM from 0.5 to 3.8 mM with flow rate 0.4 mL min^−1^; (**b**) calibration plot of peak current and glucose concentration, n = 3.

In Figure 3a, the chronoamperogram shows the current response when DMEM with various glucose concentrations sequentially flowed through the sensor. It was found that peak current increased with glucose concentration. A calibration of peak current (I) and glucose concentration in DMEM was obtained up to 3.8 mM glucose as shown in Figure 3b: I = 0.84[glucose] + 1.71, R2 = 0.99, n = 3. The limit of detection (3σ, [29]) of glucose in DMEM was 0.3 mM. The sensitivity per electrode area was 66.8 nA mM^−1^ mm^−2^.

Table 2 summarizes the performance of the reported enzymatic glucose electrodes. The developed system provided proper sensitivity for glucose in media, by which the working range was considered to be suitable for cell culture in biomedical work, e.g., proliferation and cytotoxicity assays. In order to work with cell culture in higher glucose media, in-line sample dilution could then be applied. This also helped to minimize matrix interferences such as lactate and serum protein.

### 2.3. Application

The application of the developed system was demonstrated using L929 cell line as a model. The L929 cell line is commonly used as a tool in biomedical research, such as the cytotoxicity test. In order to apply the developed SI system and sensor in L929 cell line cultivation, the amperometric measurement of the GOD–SWCNTs–AuNPs-modified SPE in 0.5–3.8 mM glucose in DMEM was repeated for 3 successive days to determine the stability of the sensor. It was observed that the glucose sensor exhibited 81–94% of current responses compared with initial values. This indicated that the sensor can be used for usual cell culture application (72 h incubation) [28].

The cell culture of L929 cell was incubated in the bioreactor for 72 h without media refresh. At 24 and 72 h after cell seeding, glucose contents were determined by two methods; the SI system (in-line analysis) and the conventional colorimetric assay based on GOD enzymatic reaction (off-line analysis).

**Figure 4:**
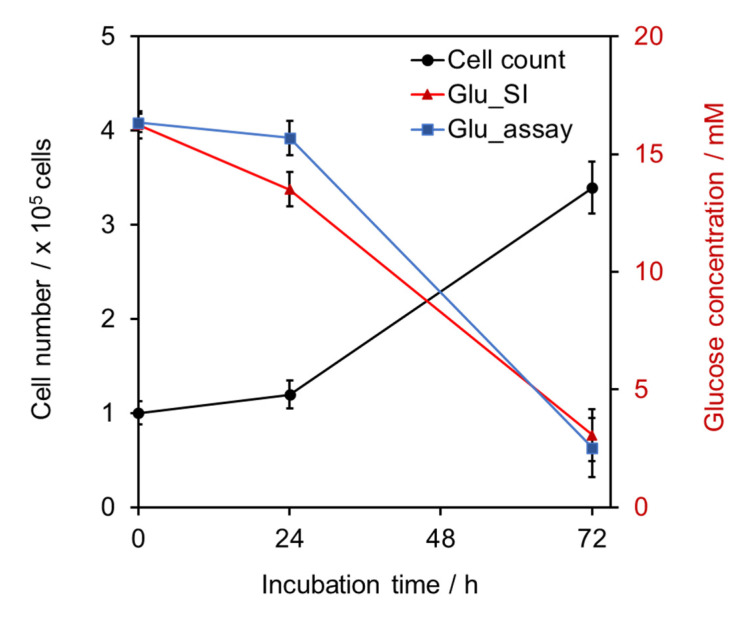
The relationship between glucose concentration in cell media determined by the developed system (red) and conventional enzymatic assay (blue) and the number of L929 cell (black) after incubated in the bioreactor for 72 h, n = 3.

Results achieved with the proposed system and the conventional colorimetric assay were in agreement (paired *t*-test, *p* > 0.05) as shown in Figure 4. The developed SI system with amperometric detection demonstrated fast, simple, and high performance compared with the conventional assay. The system could process and prepare samples, carry detection, and display a result within approximately 5 min per sample, while the conventional method required an enzyme incubation time of at least 30 min. Another advantage of the electrochemical method is that there is no interference from the color and turbidity of the culture media; for example, phenol red which is a pH indicator commonly used in many commercial media formulations can interfere with the colorimetric method. Moreover, there is no need for sample and reagent preparation for each measurement. Therefore, the SI system can reduce working time and waste generation.

Moreover, the measured glucose contents were related to the number of cells, i.e., the amount of glucose decreased as the number of cells increased. This result also suggested that the nutrient in the culture media was depleted; therefore, refreshing of the media or cell subculture was critically needed before the cell started dying. It can be developed further for an automatic culture system or a feedback control system. Therefore, this SI-bioreactor system with GOD–SWCNTs–AuNPs SPE can be employed for advanced cell culture in tissue engineering applications.

This work demonstrated the feasibility of using the developed system for monitoring metabolites in cell proliferation of L929 cell culture. It would be beneficial as a system model for applying in the study of other cell proliferation, and multiple cell monitoring in drug screening to observe the efficacy of drugs such as cancer treatment medicines. Moreover, it can be employed in multiple cell monitoring under various conditions to understand the cell behavior and its related effects in different conditions such as cell hyperglycemia and cell hyperinsulinemia that can possibly cause some diseases [30,31,32].

## 3. Materials and Methods

### 3.1. System Design and Fabrication

#### 3.1.1. The Design Concept

**Figure 5:**
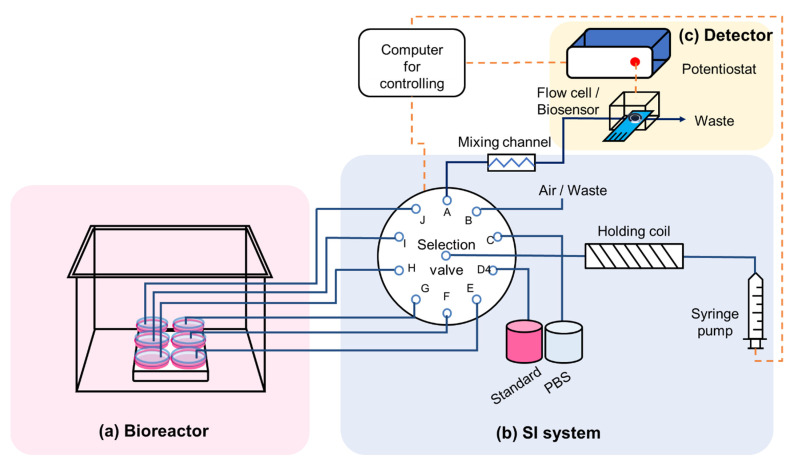
A specially designed new system for in-line glucose monitoring in cell culture application is composed of three parts: (**a**) bioreactor, (**b**) SI system, and (**c**) detector.

A specially designed system for in-line glucose monitoring in cell culture is composed of three parts: (a) bioreactor, (b) SI system, and (c) detector; as illustrated in Figure 5.

#### 3.1.2. The Bioreactor

An SI-bioreactor system was designed for in-line glucose analysis in mammalian cell culture for biomedical research in a general biological laboratory. The bioreactor is where cells are accommodated under controlled conditions. We previously reported the development of a programable automatic bioreactor for both adherent and suspension cell culture [28].

**Figure 6:**
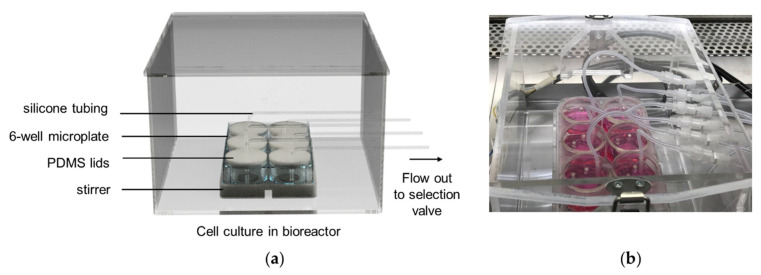
The bioreactor: (**a**) a computer-aided design (CAD) file showing components of a bioreactor; (**b**) a photograph of bioreactor with cell culture in a 6-well microplate.

In this work, the bioreactor model was modified from the previous work. The bioreactor was designed to accommodate cell culture in a standard 6-well microplate that is generally used in biological laboratory, as illustrated in Figure 6. It served as a cluster of6 units under the same incubation condition (such as temperature and CO_2_ atmosphere), but individual cell culture may be differently treated. This setup was able to monitor each bioreactor unit separately. The system was designed to be a closed system with minimal modification to a cell container in order to avoid cell stress and reduce contamination risks. The bioreactor was coupled with the SI system via a PTFE connector to create a closed system for in-line glucose analysis. Most components were sterilizable and disposable. All components that were in contact with cell or culture media were sterilized by either an autoclave, ethanol, or UV radiation, to minimize contamination.

#### 3.1.3. The SI System

The SI system was responsible for solution handling between the bioreactor and the detector unit. For in-line glucose assay in cell culture, the SI system first performed a sampling of each cell culture unit in the cluster independently. Second, the sample was in-line diluted to match the calibration graph. Before detection, a special design zigzag mixing channel was exploited instead of flow reversal operation for mixing efficiency. The sample zone ends with an air segment to minimize dispersion. The mixed solution was introduced to the electrochemical detection part without removing the air segment. These operational steps offer efficiency of the SI system and shorten operation time.

The SI system consisted of a 1-mL syringe pump (in-house assembly), a holding coil, a 10-port selection valve, a newly designed mixing channel (in-house 3D-printed), and a flow cell (in-house 3D-printed) as illustrated in Figure 5. It was connected to a laptop computer and controlled by in-house software. The software allowed both manual and pre-programed controlling. The SI sequence and volume are pre-programmable, including the in-line sample dilution step.

A mixing channel was designed in-house using a computer-aided design (CAD) program (Fusion 360, Autodesk Incorporation, San Francisco, CA, USA) and printed using an LCD-based 3D printer (Anycubic photon mono x, Hongkong anycubic technology, Hongkong, China). The acrylic resin-based 3D printing allowed precise small-scale fabrication suitable for the microfluidic platform. The design was also customizable to be able to fit with any available devices in the laboratory.

**Figure 7:**
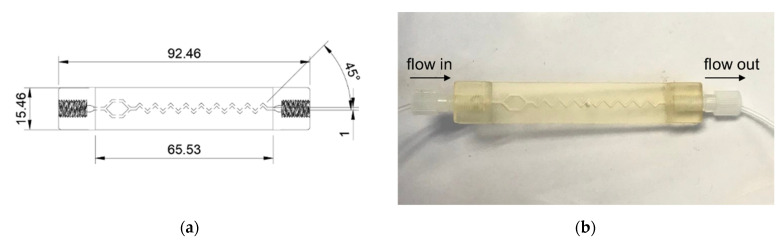
3D-printed mixing channel: (**a**) CAD design; (**b**) photograph of a mixing channel.

In Figure 7a,b, a mixing channel was designed as a 45° zigzag channel modified from previous reports [33,34]. In addition, for this system with one input channel, a Y-split part was added before the mixing channel to help mixing performance. The Y-split was designed in one to two ways with 45° and rejoined before the mixing channel. The Reynolds number was estimated and found to be 0.402 (at flow rate 0.4 mL min^−1^). The Reynolds numbers between 0.01 and 100 indicate the mixing flows are expected to be laminar, and mixing system would be highly dependent on diffusion.

#### 3.1.4. The Electrochemical Detection Unit

**Figure 8:**
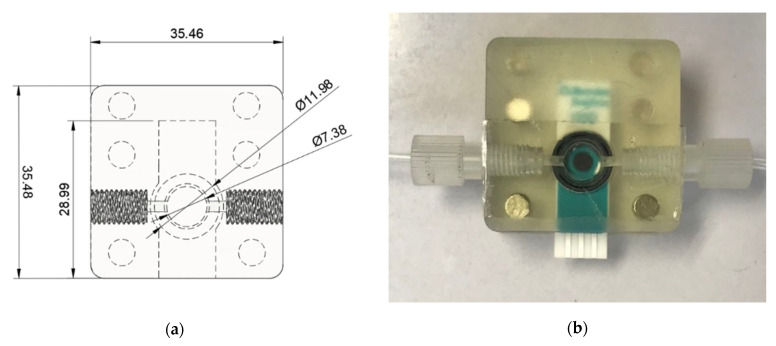
Detection unit: (**a**) CAD file of 3D-printed flow cell; (**b**) photograph of the flow cell accommodating a SPE.

For the detection unit, a flow cell was specially designed to accommodate the SPE with a flow-through chamber, as shown in Figure 8a,b. It was printed using LCD-based 3D printing, similar to the mixing channel. It allowed flow-through electrochemical measurement for a solution volume less than 100 μL. The sample was aspirated to the flow chamber at 0.4 mL min^−1^. The flow-through analysis also helped avoid biofouling on electrode by proteins.

An SPE was employed for amperometric measurement in the detection unit. It has advantages of small size, ease of use, and cost-effectiveness compared with standard three-electrode system. This makes the SPE a suitable sensor for the glucose monitoring in the SI-bioreactor system. A simple modification of commercially available, disposable SPE with GOD enzyme can be performed in a laboratory beforehand.

### 3.2. Reagents and Chemicals

All reagents and chemicals used were of analytical reagent grade. A glucose standard solution was prepared by dissolving D-(+)-glucose (C_6_H_12_O_6_, Sigma-Aldrich, Saint Louis, MO, USA) in a phosphate buffered saline (PBS) pH 7.4 (Amresco Inc, Solon, OH, USA). A glucose-free Dulbecco’s Modified Eagle Medium (DMEM) (Gibco, Thermo Fisher Scientific, Waltham, MA, USA), DMEM powder (Gibco, Thermo Fisher Scientific, USA), fetal bovine serum (FBS) (Thermo Fisher Scientific, USA), and antibiotic-antimycotic (Thermo Fisher Scientific, USA) were used without further purification.

For electrode modification, a 1000 U mL^−1^ glucose oxidase (GOD) stock solution was prepared by dissolving glucose oxidase (E.C.1.1.3.4) from *Aspergillus niger,* recombinant (269 U mg^−1^, Merck, Darmstadt, Germany) in PBS and then diluted to final concentration. A single-walled carbon nanotube (SWCNT, dispersion in H_2_O, Sigma-Aldrich, Saint Louis, MO, USA) and colloidal gold (20 nm, Kestrel Bio Science, Bangkok, Thailand) were sonicated for one hour before use. A 1% *w*/*v* chitosan solution was prepared by dissolving 1 g of chitosan oligomer (100 mesh, Taming Enterprise, Beijing, China) in 100 mL of 1% acetic acid solution and then purified by dialysis.

### 3.3. Modification of SPE

A carbon SPE (working electrode: carbon; counter electrode: carbon; reference electrode: silver) (Metrohm Dropsens, Madrid, Spain) was modified with SWCNTs, AuNPs, chitosan, and GOD. First, the SPE was activated by performing cyclic voltammetry in 0.5 M H_2_SO_4_ solution. A 10 μL aliquot of glucose oxidase solution with optimal concentration, 1 μL of 0.1 mg mL^−1^ SWCNT suspension and 1 μL of colloidal gold suspension were mixed. Later, a mixture was drop-casted on a working electrode surface and left to semi-dry. A 2 μL aliquot of chitosan solution was then drop-casted and the electrode was kept at 4 °C overnight. The SPE was rinsed with PBS thoroughly before use.

### 3.4. Electrochemical Measurement

All electrochemical measurements, cyclic voltammetry, and chronoamperometry were performed using a potentiostat (µStat 300, Dropsens, Spain) at 25 °C. The potential was reported as V vs. Ag pseudo-reference electrode.

### 3.5. Glucose Monitoring Using the System

The system was controlled by an in-house program modified from the previous study [28]. The controlling program allowed preset command script (.csv) as listed in Table 3.

**Figure 9:**
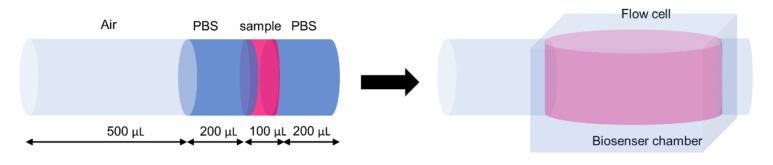
A SI sequence profile for amperometric determination of glucose in culture media.

A glucose standard solution or sample was sequentially aspirated into a holding coil to create mono-segmentation and then introduced to the detection unit where electrochemical measurement occurred. In the case of the real sample, 100 μL of culture media was sampled from the bioreactor and in-line diluted with two portions of 200 μL of PBS before detection as shown in the script in Table 4 and illustrated in Figure 9. A ratio between sample and diluent can be adjusted to obtain desired dilution factor. This also can help reduce interference from serum protein in the culture media.

### 3.6. Cell Culture of L929 Cell Line

A glucose analysis using the developed SI system was demonstrated using a cell culture of a mouse fibroblast cell line (L929: NCTC clone 929, JCRB cell bank, Osaka, Japan). The cells were maintained in a complete DMEM with 10% FBS and 1% antibiotic-antimycotic. The L929 cells were seeded in a 6-well plate at 1 × 10^5^ cells per well and incubated in the bioreactor at 37 °C with 5% CO_2_ for 72 h.

### 3.7. Glucose Enzymatic Assay

Glucose enzymatic colorimetric assay is a conventional method for the determination of glucose in a biological sample. The analysis is based on the reaction between glucose and glucose oxidase enzyme, then the released hydrogen peroxide reacts with peroxidase reagent and induces oxidative condensation of 4-aminoantipyrine and phenol to form a quinoneimine dye [35]. A total of 4 μL of GOD solution was added to 100 μL of glucose standard or sample and then the mixture was incubated at 37 °C for 30 min. A 2 μL aliquot of the incubated product was added to a 200 μL of peroxidase reagent. The absorbance of the quinoneimine dye product was measured by spectrophotometry (HiPo MPP-96, Biosan, Riga, Latvia).

## 4. Conclusions

A system consisted of bioreactor, SI system, and amperometric detector was developed for in-line glucose monitoring. The system was designed for operating six units of bioreactor for individual cell culture under different treatments. The SI system allowed automation for handling of various solutions, i.e., in-line sampling, in-line sample dilution, and mixing. The GOD–SWCNTs–AuNPs-modified SPE combined with a 3D-printed flow cell was used as a detection unit. Application on cell culture was demonstrated on fibroblast cell L929 culture.

The system can be further developed for simultaneous monitoring of a change in metabolite of cell culture with multiple cell types or simulated conditions up to six types/conditions. In addition, it can be utilized in automatic cell culture systems, such as automatic feeding of media or other solutions. The system can be applied to a wide area of biomedical research such as cellular metabolism studies for drug and cancer therapy, and in vitro cytotoxicity tests for biomaterial and tissue engineering applications.

## Figures and Tables

**Table 1:** Specification of available different enzymatic glucose sensors used in cell culture application.

Detection Technique	Sensor Components	Linear Range (mM)	Cell Type	In-Line Measurement	Ref
Optical	Oxygen sensor covered with glucose oxidase enzyme, bovine serum albumin (BSA), glycerol, glutaraldehyde	0–20	Chinese hamster ovarian (CHO) cells	Yes	[23]
Electrochemical	Multi-walled carbon nanotubes, chitosan, glucose oxidase enzyme	5–25	Human myeloid leukemia (U937) cells	Yes	[21,24]
Electrochemical (Amperometric)	Water-based carbon ink formulations containing cobalt phthalocyanine (CoPC), glucose oxidase enzyme	up to 5	Human choriocarcinoma (BeWo) cells	Yes	[25]
Electrochemical (Amperometric)	pHEMA enzyme membrane containing glucose oxidase	up to 10	Human glioblastoma multiforme (T98G) brain cancer cells	Yes	[26]
Electrochemical (Amperometric)	Carbon black–Prussian blue screen-printed electrode modified with Glucose oxidase and cellulose nanocrystals	0.1–2	NIH 3T3 fibroblast cells	No	[27]

**Table 2:** Performance of reported different enzymatic glucose electrodes.

Electrode	Sensitivity	Linear Range	Limit of Detection	Ref.
Multiwalled carbon nanotubes, chitosan, platinum nanoparticles, glucose oxidase enzyme, methyltrimethoxysilane (MTOS)	2.8 µA mM^−1^	1.2 µM−6 mM	0.3 µM	[17]
Silver nanoparticles, carbon nanotubes, chitosan, glucose oxidase enzyme	135.9 µA mM^−1^	0.5–50 µM	0.1 µM	[18]
Carbon nanotubes, gold nanoparticles, chitosan, glucose oxidase enzyme	n/a	6 µM–5 mM	3 µM	[19]
Carbon-coated tin sulfide (C-SnS) nanoparticles, glucose oxidase enzyme	439 nA mM^−1^ mm^−2^	0.03–0.7 mM	n/a	[20]
Multi-walled carbon nanotubes, chitosan, glucose oxidase enzyme	4.7 ± 1.3 nA mM^−1^ mm^−2^	5–25 mM *	1.4 mM	[21]
Water-based carbon ink formulations containing cobalt phthalocyanine (CoPC), glucose oxidase enzyme	6 nA mM^−1^	Up to 5 mM *	n/a	[25]
pHEMA enzyme membrane containing glucose oxidase	3.3 nA mM^−1^ mm^−2^	Up to 10 mM *	75 µM	[26]
Carbon black–Prussian blue screen-printed electrode modified with glucose oxidase and cellulose nanocrystals	57 ± 3 nA mM^−1^mm^−2^	0.1–2 mM	4 µM	[27]
This work	66.8 nA mM−1 mm−2	Up to 3.8 mM *	0.3 mM	

* Indicating glucose concentration in culture media.

**Table 3:** List of command in the SI system controlling program.

Command	Value	Description
Loop	Number	Set number of scripts to repeat
Pump	Number	Set volume of syringe pump to aspirate or dispense (mL)
Goto	Number	Jump to a line number that set of the script
Wait	hh:mm:ss	Set delay time
Dir	F or B	Set syringe pump direction (forward or backward)
Ch	Selection valve (A or B or C or else)	Select selection valve position
Start		Starting syringe pump
Msgbox	Text	Displays the specified text in the message box

**Table 4:** A script for glucose analysis using the SI system.

Line Number	Command	Value	Action
1	Loop	3	Repeat all actions for 3 times
2	Pump	0.5	Aspirate air 500 μL into holding coil
3	Dir	B	
4	Ch	B	
5	Start		
6	Wait	00:00:10	
7	Pump	0.2	Aspirate PBS 200 μL into holding coil
8	Dir	B	
9	Ch	C	
10	Start		
11	Wait	00:00:10	
12	Pump	0.1	Aspirate sample 100 μL into holding coil
13	Dir	B	
14	Ch	E	
15	Start		
16	Wait	00:00:10	
17	Pump	0.2	Aspirate PBS 200 μL into holding coil
18	Dir	B	
19	Ch	C	
20	Start		
21	Wait	00:00:10	
22	Pump	1	Dispense all segment 1000 μL from holding coil to sensor
23	Dir	F	
24	Ch	A	
25	Start		
26	Wait	00:00:10	
27	Pump	0.5	Aspirate air 500 μL into holding coil (cleaning)
28	Dir	B	
29	Ch	B	
30	Start		
31	Wait	00:00:10	
32	Pump	0.5	Aspirate PBS 500 μL into holding coil (cleaning)
33	Dir	B	
34	Ch	C	
35	Start		
36	Wait	00:00:10	
37	Pump	1	Dispense all segment 1000 μL from holding coil to sensor (cleaning)
38	Dir	F	
39	Ch	A	
40	Start		
41	Wait	00:00:10	
42	Goto	2	Repeat all actions for 3 times

## Data Availability

The data presented in this study are available on request from the corresponding author.
